# How to Regenerate and Protect Desert Riparian *Populus euphratica* Forest in Arid Areas

**DOI:** 10.1038/srep15418

**Published:** 2015-10-20

**Authors:** Hongbo Ling, Pei Zhang, Hailiang Xu, Xinfeng Zhao

**Affiliations:** 1 State Key Laboratory of Desert and Oasis Ecology, Xinjiang Institute of Ecology and Geography, Chinese Academy of Sciences (CAS), Urumqi 830011, China; 2 University of Chinese Academy of Sciences, Beijing 100049, China

## Abstract

We found that the most suitable flooding disturbance model for regenerating *Populus euphratica* forest was two to three times per year with a duration of 15–20 days and an intensity of 25–30 m^3^/s. The flooding should take place during the seed emergence to young tree growth stages, and should be based on flooding experiments and data from vegetation quadrats and ecological water conveyance. Furthermore, we found that tree-ring width index for *P*. euphratica declined as the groundwater depth increased, and ascertained that the minimum groundwater depths for young trees, near-mature trees, mature trees and over-mature trees were 4.0 m, 5.0–5.4 m, 6.9 m and 7.8 m, respectively. These were derived from a quantitative relationship model between groundwater depth and tree-ring width index. The range for ecological water conveyance volume was 311–320 million m^3^ in the lower reaches of the Tarim River. This study not only provides a technical basis for sustainable ecological water conveyance in the Tarim River Basin, but also offers a theoretical guide and scientific information that could be used in similar areas to regenerate and protect *Populus euphratica* around the world.

*Populus euphratica* is a rare, ancient and endangered species, and is only able to form forests in inland river basins located in arid areas^1^,^2^,^3^,^4^. It is also an essential component of desert riparian ecosystems^5^,^6^. The Forest Tree Genetic Resources Panel of the Food and Agriculture Organization of the United Nations confirmed that *P. euphratica* was a core protected tree in arid and semi-arid areas^7^,^8^. *P. euphratica* is mainly found in mid and west China, north Africa and southern Europe^9^,^10^. China has the largest area of *P. euphratica*, which accounts for 61% of the global *P. euphratica* forest^11^. Specifically, the natural *P. euphratica* forest in the Tarim River Basin, Xinjiang, accounts for 54% of the global and 89% of China’s *P. euphratica* area^8^, and represents an essential tree genetic resource^12^,^13^. Desert riparian forest mainly contains *P. euphratica* and it forms a natural eco-barrier that protects the development of oases, stabilizes river channels, maintains the ecological balance of river basins, and helps form fertile forest soil^6^,^14^,^15^. However, since the 1950 s, the use of the Tarim River water resource has become increasingly unsustainable due to climate change and intensive human disturbance^16^,^17^,^18^,^19^, and this has led to the loss of desert riparian forest in the lower reaches of Tarim River^18^,^19^,^20^. Therefore, how to regenerate and protect the damaged desert riparian ecosystem, a chief component of which is *P. euphratica*, has become a major concern of the Chinese government. In 2000, the Chinese government spent 10.7 billion yuan on the “Ecologic Water Conveyance Engineering” (EWCE) Project, whose aim was to reconstruct and protect the desert riparian *P. euphratica* forest in the lower reaches of the Tarim River.

The regeneration and protection of desert riparian *P. euphratica* forest is mainly based on river flooding disturbance and uplifting the groundwater table level^15^,^21^,^22^,^23^,^24^,^25^. There have been many previous studies on river flooding disturbance in arid lands, but most of the research has focused only on natural flooding disturbance effects on soil physics and chemical properties, distribution of plant communities, community structure, species diversity and the soil seed bank^26^,^27^,^28^,^29^. In addition, because some indices of natural flooding disturbance, such as intensity, frequency and duration, fluctuate and are volatile, simulation experiments cannot be conducted in the area^26^,^30^,^31^,^32^. Hence, previous studies have been unable to identify a suitable river flooding disturbance management plan that will help regenerate damaged ecosystems using artificial regulation (e.g., the EWCE Project in the lower reaches of the Tarim River).

Many studies^33^,^34^,^35^,^36^ have indicated that the relationship is close to streamflow and tree-ring data. Streamflow is mainly converted into groundwater to meet the ecological water requirement of vegetation^37^,^38^. Similarly, the water required by the desert riparian *P. euphratica* forest is mainly supplied by groundwater^37^. Previous research determined the minimum groundwater depth that will promote *P. euphratica* growth for the whole forest in terms of plant eco-physiological variables and the tree-ring radial stem growth of *P. euphratica* etc^8^,^39^,^40^. However, diameter at breast height (DBH) for *P. euphratica* trees is correlated with the different root distributions corresponding to different groundwater depths. Therefore, based on the results of previous studies, the EWCE Project is unable to divide water according to the different DBHs of trees using the present recommendations^8^,^39^,^40^. In addition, some studies^39^,^41^ could not accurately describe the variation in *P. euphratica* growth due to the different groundwater depths because radial stem growth is affected by a self-growth trend. Consequently, the response models based on de-growth-trend tree-ring width indices for *P. euphratica* tree-ring growth and groundwater depth are rare. According to the above analysis, this study used the ecological water conveyance data from 2000–2012 and the groundwater depth data acquired from 30 monitoring wells, to analyze the effects of different flooding disturbances on *P. euphratica* seedling growth. Firstly, we produced an optimal disturbance model for seedling growth, based on flood disturbance experiments conducted in the lower reaches of the Tarim River. Secondly, a quantitative relationship model was constructed using different groundwater depths and tree-ring width indices, and then we determined the minimum groundwater depths for different tree DBHs and developed an effective ecological water conveyance management plan to regenerate and protect desert riparian *P. euphratica* forest in the lower reaches of the Tarim River. The aims of this study were (1) to provide technical assistance that will help achieve sustainable ecological water conveyance in the Tarim River Basin, and (2) to offer scientific guidance that can be used in similar areas to regenerate and protect *P. euphratica* forest around the world.

## Materials and Methods

### Regional setting

The Tarim River Basin (34.20° ∼ 43.39°N, 71.39° ∼ 93.45°E) is located in south Xinjiang, China, and abuts the Taklamakan Desert^16^. The Tarim River is the longest inland river in China, and is surrounded by the Aksu, Yarkant, Kaxgar, Hetian, Keriya, Weigan, Kuqa, Dina, Kaidu–Kongque and Qarqan rivers^16^. The basin wide runoffs are mainly supplied by ice-melt water and precipitation, which amount to 39.83 billion m^3^ per year. The total basin area is 1.02 million km^2^, and covers five prefectures, forty-two counties and fifty-five regiments of the Construction and Production Corps of Xinjiang^20^. The population is 11.03 million.

The main part of the Tarim River is a closed watershed and has a total length of 1321 km^37^. In the past, there were nine great rivers flowing into the Tarim River. However, since the 1940 s, the Kaxgar, Weigan, Keriya, Kuqa, Dina, Kaidu–Kongque and Qarqan rivers have been cut off from the Tarim River because of climate change and human activities^20^. Only the Hetian, Yarkant, Kongque and Aksu rivers remain tributaries of the Tarim River^37^.

Both sides of the Tarim River contain desert riparian forest, mainly *P. euphratica*, which is one of the most important arid area forest vegetation communities in Asia^8^. Shrub vegetation mainly consists of *Halostachys caspica*, *Tamarix* and *Haloxylon ammodendron*, and the main herbs are *Phragmites australis*, *Alhagi sparsifolia* and *Karelinia caspica*^37^.

**Figure 1:**
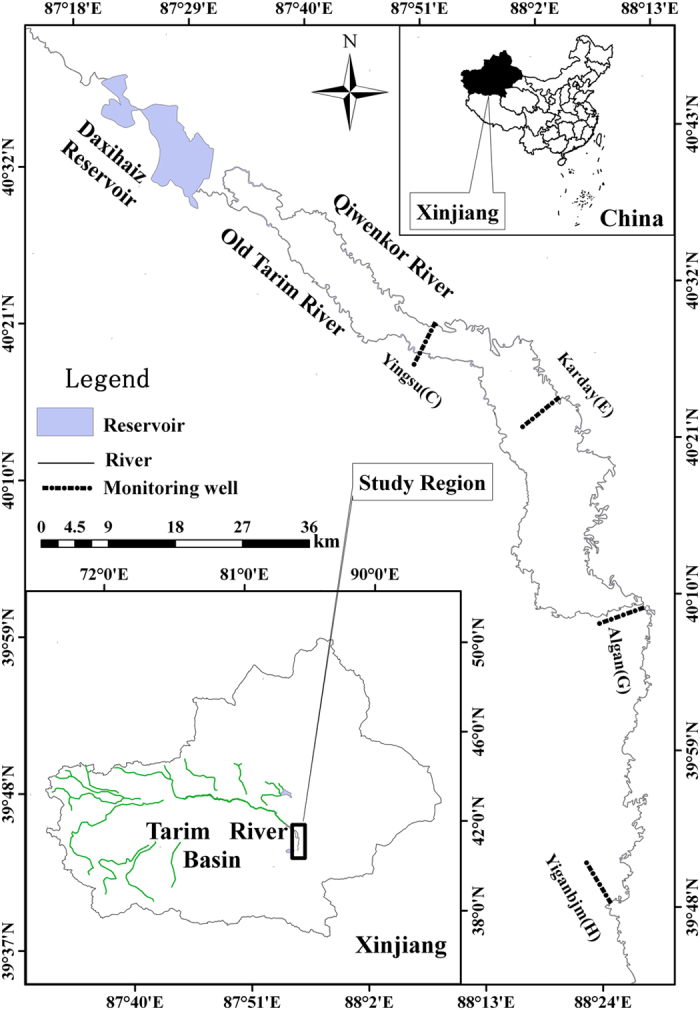
Distribution map of the four selected transects and the water conveyance channel in the lower Tarim River, China (The map was created by Hongbo ling based on the software ArcMap).

Our research area was located in the lower reaches of the Tarim River, downstream of the Daxihaizi cross-section. It covered a total area of 2191 km^2^ (Fig. 1). This area is one of the most arid areas in China with a mean annual precipitation of 40 mm and mean annual potential evaporation of 2590 mm^42^. The cumulative temperature above 10 °C for this area is 4040 to 4300 °C, with a mean diurnal range of 13 °C to 17 °C^38^.

**Figure 2:**
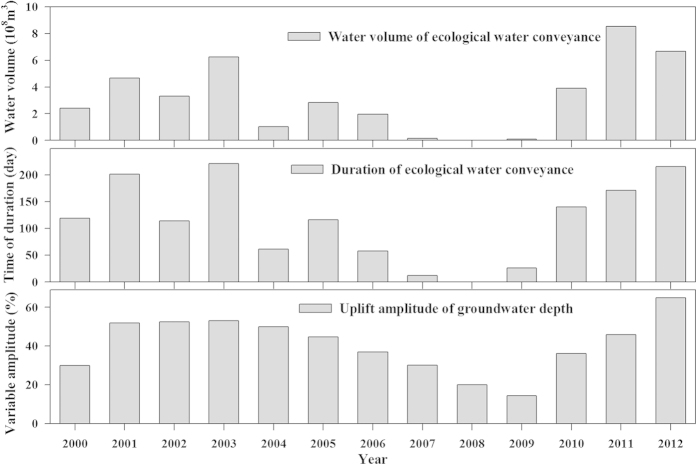
Water volume, duration and groundwater table uplifts due to increases in ecological water conveyance.

The lower reaches of Tarim River have experienced water cut offs since 1972, which has led to a reduction in the area of the desert riparian forest, a loss of *P. euphratica* forest and environmental degeneration^37^,^38^. In order to rescue the eco-environment of the lower reaches of the Tarim River, and to regenerate and protect desert riparian forest, the Chinese government, since 2000, has implemented the EWCE Project, which stretches from the Kongque River to the downstream Tarim River area^24^,^37^,^38^. From 2000 to 2006, eight times the normal water conveyance has flowed into Taitema Lake, which amounts to a total of 2.25 billion m^3^ and a mean annual amount of 0.32 billion m^3^ (Fig. 2). Between 2007 and 2009, twice the usual amount of water was allowed to flow, but because this was far short of the required amount (total amount, 20 million m^3^; mean annual amount, 10 million m^3^), the water head only reached the Kardayi Section. However from 2010 to 2012, three times the usual amount of water, which supplied 1.91 billion m^3^, at a mean annual amount of 0.64 billion m^3^, meant that water was able to resupply Taitema Lake again. As the EWCE Project proceeds, river flooding will expand to cover both sides of the river channel, which will lead to a rise in the groundwater table and help repair the damaged desert riparian forest^37^,^38^,^39^.

### Data Resources

The hydrological data used in this research were taken from the EWCE Project and the groundwater depths (the elevation minus groundwater table level) data acquired from 30 monitoring wells that were perpendicular to the river channel (the distances from wells to the river channel were 50 m, 150 m, 300 m, 500 m, 750 m and 1050 m) from 2000–2012. The vegetation data were taken from 50 flood experimental areas, 467 vegetation quadrats (Table 1) and 434 tree-ring cores from *P. euphratica* trees with different DBHs that were located in the lower reaches of the Tarim River.

### Flooding experiment

Based on the ecological water conveyance data acquired, combined with the actual river flooding conditions recorded by field investigations in the study area, we divided the flooding factors (frequency, duration and intensity) into four grades in the downstream reaches of the Tarim River (Table 2).

According to the grades, we randomly placed 3–5 vegetative quadrats that were 1 m × 1 m, 2 m × 2 m or 5 m × 5 m in size, to investigate the composition, number, height, DBH, crown width, coverage and frequency of vegetation so that we could measure changes in plant community structure under different flooding disturbance regimes. This amounted to 50 sample plots and 467 quadrats in the study area.

The results from the field research enabled us to calculate the important values (*IV*) for *P. euphratica* seedlings in each quadrat so that we could determine whether the *P. euphratica* seedlings would survive or not.


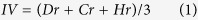


where *Dr* represents relative density; *Cr* represents relative coverage; and *Hr*. represents relative height^43^,^44^.

### Sampling and processing of tree rings

Four transects where *P. euphratica* predominated were selected as the study region, i.e., Yingsu (C), Karday (E), Algan (G) and Yiganbujima (I) (Fig. 1). We chose six to ten trees with different DBHs around one monitoring well in order to reduce the deviation. According to the principles of the International Tree-ring Database, we selected 217 trees and 434 tree-ring cores between August, 2009 and August, 2013., Based on the field survey and tree-ring analysis of *P. euphratica* forest in the lower reaches of Tarim River, the 217 trees were divided into five grades according to DBHs so that we could study the different *P. euphratica* growth periods. These were 4–10 cm (young trees), 10–30 cm (near-mature trees), 30–40 cm (mature trees) and >40 cm (over-mature trees) (Table 3). The tree-ring sampling and cross-dating were completed according to the standards published by Fritts^45^. Two cores were taken from each tree using an increment borer.

The samples were handled according to dendro-chronological laboratory procedure (i.e., drying, mounting, sanding and primary cross-dating)^45^,^46^. Firstly, the ring increments of *P. euphratica* were measured using the LINTAB^TM6^ system (precision of 0.001 mm; Rinntech, Heidelberg, Germany). Secondly, cross-dating of increment series data was determined using COFECHA software^47^. Finally, to establish standard and residual chronologies, the growth trend curve for the raw ring increments of *P. euphratica* was fitted using the negative exponential formula in the ARSTAN software^45^,^48^.

### Calculation of the tree-ring width index

In extreme arid lands (e.g., Tarim River Basin), groundwater and tree age are the two most critical factors influencing the tree-ring width of *P. euphratica*. In order to highlight the effect of groundwater on radial stem growth, we needed to remove the influence of tree age. Therefore, a standardized protocol for radial stem growth data was adopted, i.e., the raw ring growth value (*w*_*i*_), measured using COFECHA software, was divided by the expected value (*y*_*i*_) calculated from the growth trend curve by the negative exponential formula in the ARSTAN software. This produced a new sequence for the tree-ring width index (*I*_*i*_), as shown in formula 2^49^.


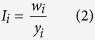


### Monotony trend test and abrupt change test for tree-ring width index

The trend change for the tree-ring width index was analyzed using the Mann-Kendall monotony trend test. In the test process^39^,^50^, when the statistic (i.e., *Z*_*c*_) is positive, it reflects an upward trend; whereas when *Z*_*c*_ is negative, it reflects a downward trend. If the checking standards are salient (|*Z*_c_| ≥ |*Z*_0.05_| = 1.96 at the 95% level; |*Z*_c_ | ≥ |*Z*_0.01_| = 2.58 at the 99% level), then the trend is credible, otherwise it is not.

To analyze abrupt change, we first calculated the turning point of the tree-ring width index and groundwater depth using the accumulated anomaly value^41^. Then we checked whether the abrupt changes in the tree-ring width indices either sides of the turning point were salient according to the Mann-Whitney abrupt change test^39^,^51^. If the statistic (|*Z*_c_ |) > 1.96, then the abrupt change is significant, otherwise it is not.

## Results

### The most suitable river flooding disturbance model for *P. euphratica* seedlings (DBH < 4 cm)

**Figure 3:**
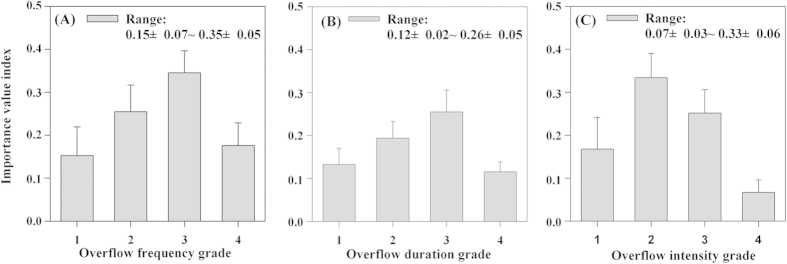
The importance values *(IV*) for *Populus euphratica* seedlings under different disturbance patterns.

The importance value (*IV*) can show the most suitable habitat for this species^52^. Based on the field trial results, we divided the 467 quadrats into different grades according to the disturbance patterns, calculated the *IV* value for *P. euphratica* seedlings (Fig. 3) and identified the most suitable flooding disturbance model for *P. euphratica* seedling regeneration.

According to Fig. 3A, as the frequency of flooding disturbance increases, the *IV* value shows a para-curve trend with the highest value appearing in Grade 3 (two to three times per year). When the overflow duration (Fig. 3B) is <15–20 days (i.e., Grade 3), then the *IV* value is relatively high and Grade 3 is the highest, but over 20 days, the value drops significantly (*P* < 0.001). Figure 3C shows that Grade 2 (25–30 m^3^/s) has the highest value and that the value declines significantly when the flood intensity is over 40 m^3^/s (*P* < 0.001). Therefore, the most suitable flooding disturbance model is two to three times per year with a duration of 15–20 days and an intensity of 25–30 m^3^/s.

### The effect of groundwater depth on young *P. euphratica* trees (DBHs 4–10 cm)

**Figure 4:**
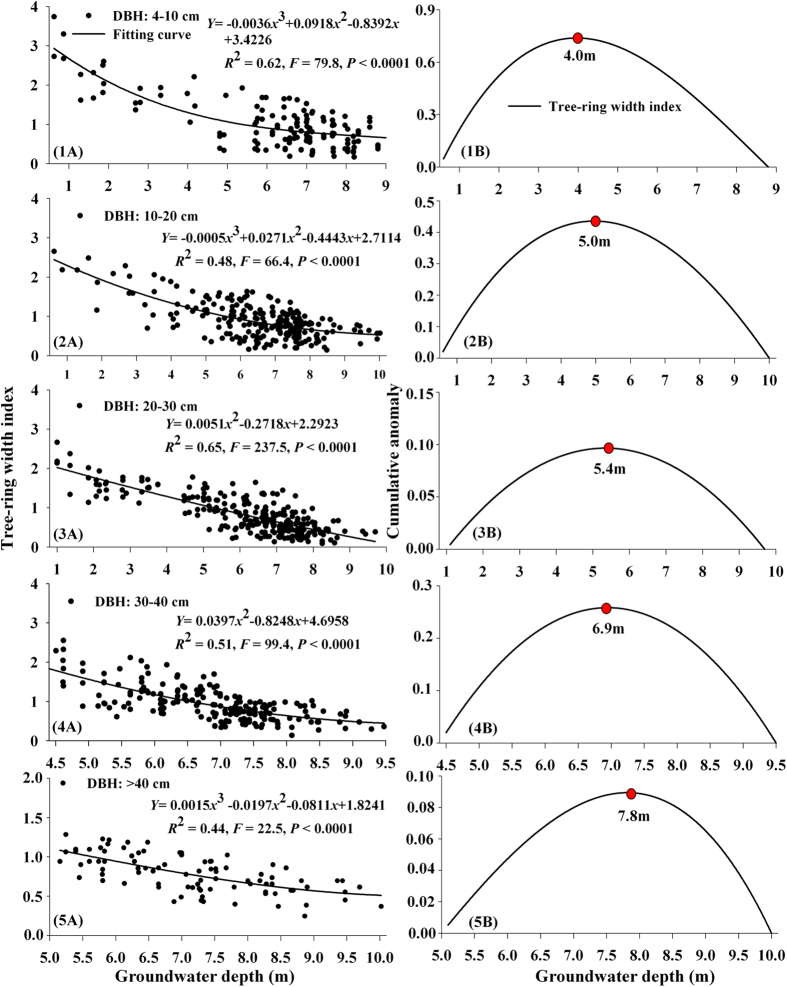
The relationships between the tree-ring width indexes for five grade DBHs and the groundwater depths (**1A–5A**), and the most suitable groundwater depths for *P. euphratica* (**1B–5B**).

Flooding disturbance (or overflow) is the key factor affecting *P. euphratica* seedling survival^53^,^54^. A suitable groundwater depth is another essential environmental factor that ensures that seedlings can grow into young trees^55^. However, trees with different DBHs respond to groundwater depth differently. Therefore, we divided the trees into five growth groups (i.e., seedling, young trees, near-mature trees, mature trees and over-mature trees) according to their DBHs. Based on the tree-ring width and groundwater depth data, we constructed a quantitative model that included tree-ring width index and groundwater depth and determined how tree-ring width index responded to groundwater depth (Fig. 4).

Figure 4(1A) shows that the tree-ring width index for young trees declines as the groundwater depth increases (*R*^2^ = 0.62, *P* < 0.0001), which means that we can use the tree-ring index to deduce the most suitable groundwater depth. In addition, according to the Mann-Kendall monotony trend test (Table S1), when groundwater depth is between 0.6 to 8.8 m, the test statistics for tree-ring width index decrease significantly (Zc is −13.32 , |*Z*_c_| > |*Z*_0.01_| = 2.58). This shows that *P. euphratica* growth is very sensitive to variation in groundwater depth. We can also calculate the decrease in tree growth for every 0.1 m increase in groundwater depth. Figure 4(1B) shows that the 4.0 m depth mark divides the curve into two parts: a left part (groundwater depth between 0.6 and 4.0 m) and a right part (groundwater depth between 4.1 and 8.8 m). When the groundwater depth is between 0.6 and 4.0 m, the average tree-ring width index is higher than the total average, which indicates a sharp decrease. When the groundwater depth is between 4.1 and 8.8 m, the average is lower than the total average, which means the tree-ring width index decreases slowly. According to the Mann-Whitney abrupt change test (Table S1), between the two groundwater depth intervals (0.6–4.0 m and 4.1–8.8 m), the mean value for tree-ring width index drops from 0.049 to 0.013, and its abrupt change is significant at the 0.01 test level. (Zc = –7.75, |*Z*_c_| > |*Z*_0.01_| = 2.58). When the groundwater depth is <4 m, the decrease in *P. euphratica* radial stem growth is relatively small and the tree-ring width index response to groundwater depth becomes less sensitive, which means that *P. euphratica* growth is under stress. Thus, the minimum groundwater depth for young trees should be <4.0 m.

### The effect of groundwater depth on near-mature *P. euphratica* trees (DBHs of 10–30 cm)

*P. euphratica* trees with DBHs of 10–30 cm grew well, with lots of newly bursting branches, and could be described as near-mature trees. In order to efficiently identify the water resource need to protect these near-mature trees, we divided the trees into two grades according to DBH (10–20 cm and 20–30 cm).

Figure 4(2A) shows that the tree-ring width index for young trees declines as the groundwater depth increases and this is significantly negatively correlated (*P* < 0.01). According to the Mann-Kendall monotony trend test (Table S2), the tree-ring width index declines significantly at the 0.01 test level (Zc = −14.35, |*Z*_c_| > |*Z*_0.01_| = 2.58). Figure 4(2B) shows that the turning point for the decreasing tree-ring width index is 5.0 m. In addition, the Mann-Whitney abrupt change test results (Table S2) for the two groundwater depth intervals (0.6–5.0 m and 5.1–8.8 m) show that the mean value for tree-ring width index drops significantly (test statistic (Zc) is −8.39) from 0.031 to 0.013. When the groundwater depth is >5.0 m, the tree-ring width index (trees with DBHs 10–20 cm) response to groundwater depth becomes less sensitive. Therefore, the most suitable groundwater depth for near-mature trees (with DBHs 10–20 cm) should be <5.0 m.

Subsequently, we constructed a response function for tree-ring width index against the variation in groundwater depth based on the data for near-mature tree-rings (trees with DBHs 20–30 cm) and groundwater depth (Figs 4(3A,3B)).

Figure 4(3A) shows that there is a significant negative correlation between tree-ring width index for near-mature trees (DBHs 20–30 cm) and groundwater depth (*R*^2^ = 0.62, *P* < 0.0001). According to the Mann-Kendall monotony trend test (Table S3), increasing groundwater depth leads to a significant decrease in tree-ring width index (Zc = −13.71, |*Z*_c_| > |*Z*_0.01_| = 2.58). Figure 4(3B) and Table S3 show that the turning point is 5.4 m, and between the two groundwater depth intervals (1.1–5.4 m and 5.5–9.7 m), the mean tree-ring width index value significantly drops (|*Z*_c_| = 8.03 > |*Z*_0.01_| = 2.58) at the 0.01 test level from 0.024 to 0.019. Therefore, we can conclude that the minimum groundwater depth for near-mature trees (DBHs 20–30 cm) is 5.4 m. In brief, the sensitive interval for near-mature tree (DBHs 10–30 cm) response to groundwater depth is between 5.0 to 5.4 m, and the most suitable groundwater depth is <5.4 m.

### The effect of groundwater depth on *P. euphratica* mature trees (DBHs 30–40 cm)

Mature *P. euphratica* trees are very important components of the *P. euphratica* community in the Tarim River Basin. Therefore, we studied the growth response of mature trees to different groundwater depths (Fig. 4(4A,4B) and Table S4).

According to Fig. 4(4A), there is a negative correlation between tree-ring width index for mature trees and groundwater depth (*R*^2^ = 0.51, *P* < 0.0001). The Mann-Kendall monotony trend test (Table S4) shows that the tree-ring width index holds a significant decrease trend (Zc = −10.35 > |*Z*_0.01_| = 2.58). Figure 4(4B) shows that the turning point is 6.9 m. The Mann-Whitney abrupt change test (Table S4) between the two groundwater depth intervals (<6.9 m and >6.9 m), shows that the mean value for tree-ring width index significantly declines from 0.038 to 0.017, (test statistic Zc = −6.12). Based on the results above, we can conclude that the minimum groundwater depth for mature trees (DBHs 30–40 cm) is 6.9 m and the most suitable groundwater depth should be <6.9 m.

### The effect of groundwater depth on *P. euphratica* over-mature trees (DBHs > 40 cm)

In the lower reaches of the Tarim River, *P. euphratica* over-mature trees are mainly found in areas that are more than 1 km from the river. At this distance, the recharge ability of the river is weak and the groundwater table is low. However, the over-mature trees can resist sandstorms and protect the stability of the natural vegetation ecosystem on both sides of the river. Therefore, we analyzed the correlation between the growth of over-mature trees and groundwater depth (Fig. 4(5A,5B) and Table S5).

Figure 4(5A) shows that there is a significant negative correlation between tree-ring width index for over-mature trees and groundwater depth (*R*^2^ = 0.44, *P* < 0.0001). The Mann-Kendall monotony trend test results (Table S5) show that an increase in groundwater depth leads to a significant decrease in tree-ring width index (|*Z*_c_| = 10.24 > |*Z*_0.01_| = 2.58). Therefore there is a considerable correlation between the two results. Figure 4(5B) shows that the turning point is 7.8 m for the tree-ring width index. The Mann-Whitney abrupt change test results (Table S5) for the two groundwater depth intervals (<7.8 m and >7.8 m) show that the mean value for the tree-ring width index significantly drops (test statistic (Zc) is −6.02) from 0.015 to 0.007. Thus we can conclude that the minimum groundwater depth for over-mature trees (DBHs >40 cm) is 7.8 m.

## Discussion

### The regeneration process for *P. euphratica* seedlings

The intermediate disturbance hypothesis^56^,^57^ is one of the most significant theories in ecology. This theory can be used to guide ecological protection and natural resource planning, and improve the regeneration of *P. euphratica* forest because river flooding disturbance can activate the soil seed bank and accelerate the regeneration of vegetation^24^.

*P. euphratica* seeds are smaller than most shrub seeds, and the radicles and plumules after germination are weaker than those of perennial herbs, so they do not compete well for water and have a low water storage capacity. Our results suggest that we should flood the area 2–3 times per year during the seed germination to seedling growth. However, over-frequent flooding is harmful for *P. euphratica* seedling establishment, and causes the *IV* value to decline significantly when the frequency of flooding is 4–5 times per year.

Based on the ecological water conveyance data, a minimum flow velocity of 20 m^3^/s can maintain the hydrological process in the lower reaches of the Tarim River, but an intensity below 20 m^3^/s does not lead to overflow. However, when the flow velocity is above 35 m^3^/s, the scouring effect of the overflow on the river bed becomes severe and *P. euphratica* seedlings are barely able to establish and survive.

Our field research results showed that water-loving plants (such as *Phragmites australis* and *Apluda mutica* Linn.) are prevalent in areas where the flooding duration is over 30 days. However, we do not consider that the flora in the lower reaches of the Tarim River represents a climax community, as the ecosystem is constrained by poor water conditions. In contrast, *P. euphratica* is an important climax species in desert riparian forest, and can be adapted to the arid environment in the Tarim River Basin^58^. In addition, some studies^24^,^59^,^60^ have shown that long term surface water will raise the groundwater depth and lead to salt accumulation in the surface soil. *P. euphratica* seedling growth requires a low salt environment, and if the salinity of the soil is high, seedling growth will be suppressed^61^. Therefore, the *IV* value declines significantly when the surface water duration is over 20 days. Our quantitative results show that the most suitable flood duration is 15–20 days, which is more practicable and achievable when attempting to regenerate *P. euphratica* forest in downstream areas of the Tarim River.

### Minimum groundwater depth for desert riparian *P. euphratica* forest

The growth of *P. euphratica* is close correlated with groundwater depth^24^. As the groundwater depth increases, survival and growth rates of *P. euphratica* decline and the trees become less sensitive to groundwater depth. Previous research^8^,^62^ showed that when the groundwater depth was >4.5 m, the significance of groundwater to *P. euphratica* growth becomes less important; the net photosynthetic rate and transpiration rate change, the proline and abscisic acid (ABA) contents become abnormal and the growth of *P. euphratica* is restricted. When the groundwater depth is >8 m, the trees begin to seriously decay or even die. According to previous study^39^, the correlation between radial stem growth and groundwater depth shows that the minimum groundwater interval is between 4.71 m and 8.62 m. In reality, our results suggested that the most suitable groundwater depth is <4 m. The results of this study also indicate that the minimum groundwater range is between 4.0 m and 7.8 m (i.e., young trees, 4.0 m; near-mature trees, 5.0–5.4 m; mature trees, 6.9 m; over-mature trees, 7–8 m), which is similar to previous research results. However, the most significant result in our study is that the minimum groundwater depths are confirmed for *P. euphratica* with different DBHs. In the future, the ecological water conveyance, through the use of the EWCE and the ecological water amount, can be defined according to the distribution features of *P. euphratica* forest and groundwater depth, Water is first transported to *P. euphratica* forest via deep groundwater. Therefore, this study will provide a theoretical basis and scientific guidance for the rational utilization of water resources and regeneration of *P. euphratica* forest in arid areas.

Our results show that the decrease in tree-ring width index becomes less sensitive to tree-ring growth as groundwater depth increases. The reason is that when *P. euphratica* is suffering from water stress, the tree reduces transpiration by secreting ABA to regulate stomatal conductance, which inhibits tree growth^62^.

### The best ecological water conveyance levels for *P. euphratica* protection in the lower reaches of the Tarim River

Based on the best flooding disturbance model (three times a year, duration 20 days and intensity 30 m^3^/s), the calculated ecological water amount needed is 160 million m^3^. The data for ecological water conveyance between 2000 and 2012 in the lower reaches of the Tarim River showed that the annual average water volume was 320 million m^3^. Therefore, it can meet the water requirements of the flooding disturbance model for *P. euphratica* seedlings.

**Figure 5:**
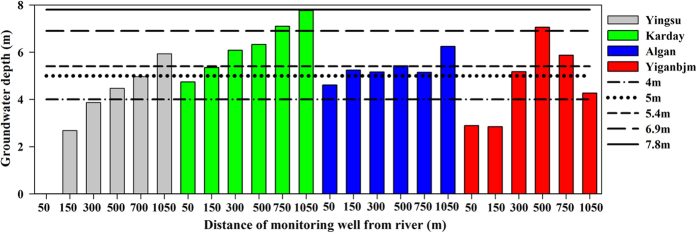
Groundwater depths at different distances from the river and the minimum groundwater depth requirements for *P. euphratica* trees.

Our results also show that the groundwater depth should be shallower than the minimum groundwater depth (young trees, 4.0 m; near-mature trees, 5.0–5.4 m, mature trees, 6.9 m and over-mature trees, 7.8 m) to ensure that *P. euphratica* trees grow well. Figure 5 shows that the groundwater depth in the four transects (Yingsu, Karday, Algan and Yiganbjm) have a strong relationship with the minimum groundwater depth for *P. euphratica* with different DBHs. The recorded data from the four transects shows that young trees (DBHs 4–10 cm) are mainly found within 50 m of the channel and the majority is located in Algan. The near-mature trees (DBHs 10–30 cm) are mainly found 150 m from the channel and mature trees (DBHs 30–40 cm) and over-mature trees (DBHs >40 cm) are mainly located 500 m and 1000 m away from the channel. Figure 5 also shows that the actual groundwater depths are higher than the minimum groundwater depth. According to a previous study result^63^, the ecological water requirement for maintaining *P. euphratica* forest transpiration and minimum groundwater depths is 311 million m^3^. In view of water shortages in this river basin^18^,^37^, and the requirements of ecological water and river flooding in the lower reaches, the most suitable range of ecological water conveyance is 311–320 million m^3^, which should be sufficient to regenerate and protect *P. euphratica* in the lower reaches of the Tarim River.

## Conclusion

### Our results show that

1. During the seed to young tree growth period, the most suitable flooding disturbance model for *P. euphratica* regeneration is 2–3 times per year with a duration of 15–20 days and an intensity of 25–30 m^3^/s.
2. The tree-ring width index for *P. euphratica* significantly declines as the groundwater depth increases.
3. The minimum groundwater depths needed for *P. euphratica* young trees, near-mature trees, mature trees and over-mature trees are 4.0 m, 5.0–5.4 m, 6.9 m and 7.8 m, respectively. In order to protect the desert riparian forest, the actual groundwater depth should be smaller than the minimum groundwater depth for each tree growth stage.
4. The most suitable range for water conveyance amount is 311–320 million m^3^, and this meets the requirements for optimal flooding and ecological water for *P. euphratica* growth in the lower reaches of Tarim River.

## Additional Information

**How to cite this article**: Ling, H. *et al*. How to Regenerate and Protect Desert Riparian *Populus euphratica* Forest in Arid Areas. *Sci. Rep*. **5**, 15418; doi: 10.1038/srep15418 (2015).

## Supplementary Material

Supplementary Information

## Figures and Tables

**Table 1:** Vegetative investigation of the quadrats for flooding experiment in the lower reaches of Tarim River.

Number	Specie	Genera	Family	Age
1	*Populus euphratica* Oliv.	*Populus* L.	Salicaceae	1-3a
2	*Tamarix ramosissima* Ledeb.	*Tamarix* L.	Tamaricaceae	1-3a
3	*Tamarix hispida* Willd.	*Tamarix* L.	Tamaricaceae	1-3a
4	*Tamarix hohenacheri* Bunge.	*Tamarix* L.	Tamaricaceae	1-3a
5	*Tamarix elongata* Ledeb.	*Tamarix* L.	Tamaricaceae	1-3a
6	*Tamarix laxa* Willd.	*Tamarix* L.	Tamaricaceae	1-3a
7	*Alhagi sparsifolia* Shap.	*Alhagi Gagneb*.	Fabaceae	1-2a
8	*Halimodedron halodendron* Voss	*Halimodendron Fisch. ex DC*.	Fabaceae	1-2a
9	*Glycurrhiza inflata* Bat.	*Glycyrrhiza* L.	Fabaceae	1-2a
10	*Sophora alopecuroides* L.	*Sophora* L.	Fabaceae	1-2a
11	*Halostachys caspica* C. A. Mey.	*Halostachys* C. A. Mey.	Chenopodiaceae	1-2a
12	*Halocnemum strobiaceum* Bieb.	*Halocnemum* Bieb.	Chenopodiaceae	1-2a
13	*Salsola pellucida* Litv.	*Salsola* L.	Chenopodiaceae	1a
14	*Salsola ruthenica* Iljin.	*Salsola* L.	Chenopodiaceae	1a
15	*Halogeton glomeratus* C. A. Mey.	*Salsola* L.	Chenopodiaceae	1a
16	*Suaeda prostrata* Pall.	*Suaeda* Forsk. Ex Scop.	Compositae	1-2a
17	*Halogeton arachnoideus* Moq.	*Saliconia* L.	Compositae	1a
18	*Karelinia caspica* Less.	*Karelinia* Less.	Compositae	1-2a
19	*Yang Hexinia polydichotoma* H. L. Yang.	*Hexinia* H. L. Yang	Compositae	1-2a
20	*Inula salsoloides* Ostenf.	*Inula* L.	Compositae	1-2a
21	*Taraxacum* sp.	*Taraxacum* Weber	Compositae	1-2a
22	*Acroption repens* Prodr.	*Acroptilon* Cass.	Compositae	1-2a
23	*Scirpus planiculmis* Fr. Schmidt	*Scirpus* L.	Cyperaceae	1-2a
24	*Scirpus tabernaemontani* C. C. Gmel.	*Scirpus* L.	Cyperaceae	1-2a
25	*Aeluropus pungens* Var.	*Aeluropus* Trin	Gramineae	1-2a
26	*Phragmites australis* Trin.	*Phragmites* Trin	Gramineae	1-2a
27	*Apocynum venetum* L.	*Apocynum* L.	Apocynaceae	1-2a
28	*Lycium ruthenicum* Murr.	*Lycium* L.	Solanaceae	1-2a
29	*Elaeagnus angustifolia* Linn.	*Elaeagnus*	Elaeagnaceae	1-3a
30	*Equisetum arvense* L. Sp. pl	*Hippochaete* Milde	Equisetaceae	1-2a
31	*Cynanchum sibiricum* Willd.	*Cynanchum* L.	Asclepiadaceae	1-2a

**Table 2:** Gradient divisions for three flooding factors at monitoring sections in the Lower Tarim River.

Grade	Flooding frequency	Flooding duration (day)	Flooding intensity (m^3^/s)
1	once in one or two years	5–10	20–25
2	once or twice per year	10–15	25–30
3	2–3 times per year	15–20	30–35
4	4–5 times per year	>20	35–40

**Table 3:** Classification standards of five grades for *Populus euphratica* in the lower Tarim River.

DBH(cm)	Growth stage	Age(year)	Growth characteristics
4–10	Young trees	5–15	Branches grow broad leaves in the upper canopy, and narrow leaves in the lower canopy. The trees have no characteristics of blossom and fruit setting.
10–30	Near-mature trees	15–40	Braches grow broad leaves in the canopy.A small amount of new braches are germinated in the tree trunk. The trees begin to bloom and bear fruit.
30–40	Mature trees	40–60	Braches grow broad leaves in the canopy. No or few branches wither up. The trees are at the peak period of blossom and fruit setting.
>40	Over-mature trees	>60	Braches grow broad leaves in the canopy.A large number of branches wither up.The trees bear little or no fruit.
