# Supplementary material for: How to Regenerate and Protect Desert Riparian Populus euphratica Forest in Arid Areas
Source: Sci Rep. 2015 Oct 20;5:15418. doi: 10.1038/srep15418 (PMC4612519; doi:10.1038/srep15418)
Supplement: Supplementary Information [file srep15418-s1.pdf]

Supplementary information

How to Regenerate and Protect Desert Riparian *Populus euphratica* Forest in Arid Areas

Hongbo Ling [1,2], Pei Zhang [1,2], Hailiang Xu [1], Xinfeng Zhao[1]

[1]State Key Laboratory of Desert and Oasis Ecology, Xinjiang Institute of Ecology and Geography, Chinese Academy of Sciences (CAS), Urumqi 830011, China

[2] University of Chinese Academy of Sciences, Beijing 100049, China

Table S1. The monotony trend and abrupt change test values for tree-ring width index at different groundwater depths

Item	Test method	Groundwater depth (m)	Mean value	Standard deviation	Zc	H_0	Trend
Tree-ring index	Mann-Whitney	0.6~4.0	0.049	0.014	–7.75	*R*	Decrease
		4.1~8.8	0.013	0.007			
	Mann-Kendall	0.6~8.8	0.028	0.021	–13.32	*R*	Decrease

Note: *R*-rejected, *A*-accepted.

Table S2. The monotony trend and abrupt change test results for tree-ring width index at different groundwater depths (trees with DBHs 10–20 cm)

Item	Test method	Groundwater depth (m)	Mean value	Standard deviation	Zc	H_0	Trend
Decrement of tree-ring index	Mann-Whitney	0.6~5.0	0.031	0.006	–8.39	*R*	Decrease
		5.1~10	0.013	0.005			
	Mann-Kendall	0.6~10	0.021	0.011	–14.35	*R*	Decrease

Note: *R*-rejected, *A*-accepted.

Table S3. The monotony trend and abrupt change test results for the tree-ring width index at different groundwater depths (DBHs 20–30 cm)

Item	Test method	Groundwater depth (m)	Mean value	Standard deviation	Zc	H_0	Trend
Tree-ring index	Mann-Whitney	1.1~5.4	0.024	0.001	–8.03	*R*	Decrease
		5.5~9.7	0.019	0.001			
	Mann-Kendall	1.1~9.7	0.022	0.003	–13.71	*R*	Decrease

Note: *R*-rejected, *A*-accepted.

Table S4. The monotony trend and abrupt change test results for the tree-ring width index at different groundwater depths (mature trees)

Item	Test method	Groundwater depth (m)	Mean value	Standard deviation	Zc	H_0	Trend
Tree-ring index	Mann-Whitney	4.5~6.9	0.038	0.006	–6.12	*R*	Decrease
		7.0~9.5	0.017	0.006			
	Mann-Kendall	4.5~9.5	0.027	0.012	–10.35	*R*	Decrease

Table S5. The monotony trend and abrupt change test results for the tree-ring width index at different groundwater depths (over-mature trees)

Item	Test method	Groundwater depth (m)	Mean value	Standard deviation	Zc	H_0	Trend
Tree-ring index	Mann-Whitney	5.1~7.8	0.015	0.002	–6.02	*R*	Decrease
		7.9~10	0.007	0.003			
	Mann-Kendall	5.1~10	0.011	0.004	–10.24	*R*	Decrease
